# Eye movement patterns in infants suggest illusory motion perception induced by stationary gradation

**DOI:** 10.1038/s41598-018-20865-5

**Published:** 2018-02-28

**Authors:** Soyogu Matsushita, Kazuki Sato, Kosuke Murakami, Shuma Tsurumi, So Kanazawa, Masami K. Yamaguchi

**Affiliations:** 1 Graduate School of Human Sciences, Osaka University, Osaka, Japan; 2 Department of Psychology, Chuo University, Tokyo, Japan; 3 Japan Society for the Promotion of Science, Tokyo, Japan; 4 Department of Psychology, Japan Women’s University, Tokyo, Japan; 5 Present Address: Osaka Shoin Women’s University, Osaka, Japan

## Abstract

Infants less than 1 year old are known to preferentially look at pictures of motion illusion induced by luminance gradation. However, the mechanisms underlying infant’s perception of motion illusion remain unclear. The current study analyzed the eye movement patterns of infants perceiving a motion illusion induced by stationary luminance gradations (a derivative of the Fraser-Wilcox illusion). Infants produced the same movement patterns that increase the magnitude of illusory motion in adults. We conclude that infants and adults similarly perceive motion illusion.

## Introduction

Infants perceive not only geometrical illusions such as the Ebbinghaus illusion^1^ but also the motion illusion induced by luminance gradation^2^. Kanazawa *et al*.^2^ reported that 6- to 8-month-old infants preferentially looked at a motion illusion induced by a luminance gradient in stationary patches (Kitaoka’s “Rotating Snake” illusion). However, the mechanisms underlying infant visual motion illusion perception remain unclear.

**Figure 1:**
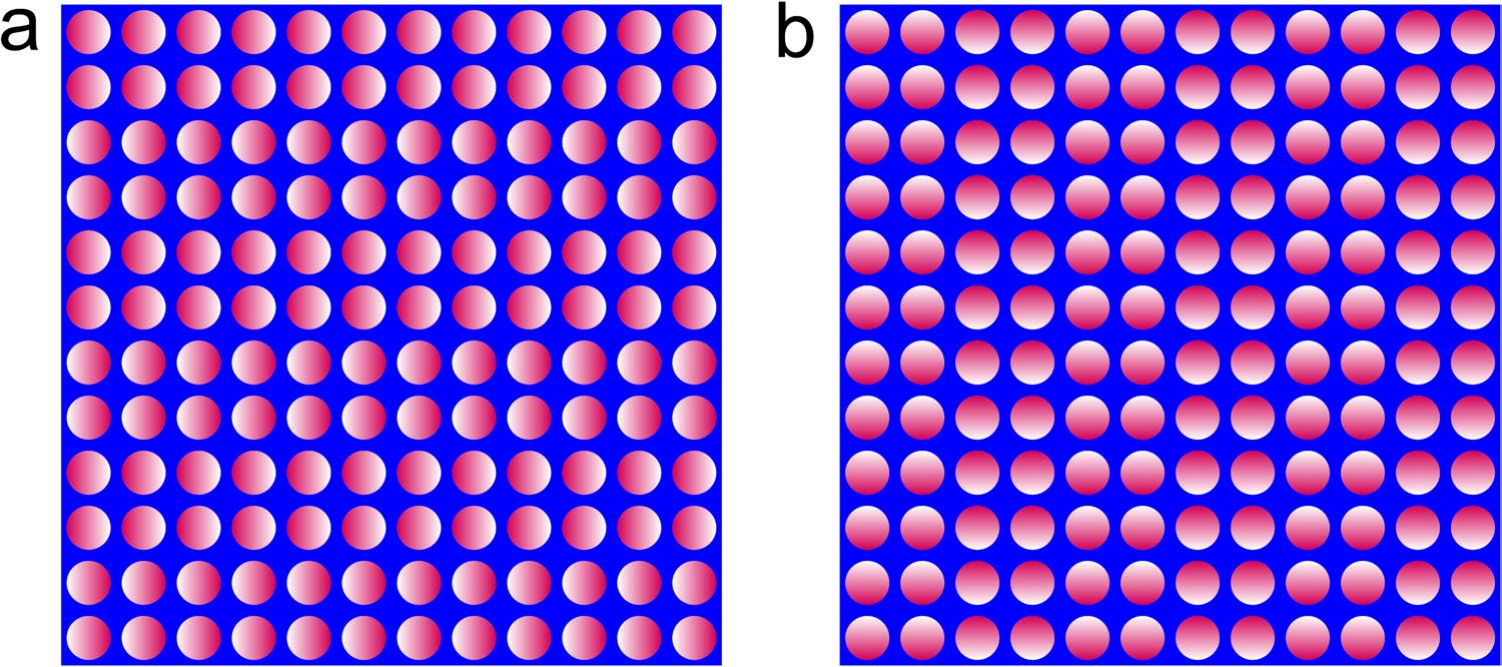
Horizontal illusion stimulus supposed to induce horizontal motion perception (**a**) and Vertical illusion stimulus supposed to induce vertical motion perception (**b**).

The current study analyzed the eye movement patterns of infants perceiving a motion illusion induced by stationary luminance gradations (Fig. 1; hereafter referred to as the “gradation-motion illusion”). This figure is a derivative of the Frazer-Wilcox illusion and the same as the Rotating Snake, which has patches that appear to move in the luminance shift direction (e.g., white to magenta) to most adults. Recently, Matsushita and colleagues reported two findings regarding eye movements during the gradation-motion illusion in adults^3,4^. First, the magnitude of illusory motion was significantly greater when the direction of the saccade and luminance gradation were orthogonal than when they were parallel. Second, participants’ eye movements tended to be orthogonal rather than parallel with respect to gradation direction while perceiving the illusory figure under conditions of unrestricted eye movement. In the current study, we examined whether infants exhibit a similar orthogonal eye movement pattern to that of adults, which would indicate a similar kind of illusory perception.

## Method

### Participants

Twenty-one infants, aged 173 to 249 days old (*M* = 206.35, *SD* = 25.47), participated in the study. This experiment was approved by Ethical Committee of Chuo University. The study was conducted in accordance with the Declaration of Helsinki. Informed consent was obtained from the parent of each infant.

### Stimuli and Apparatus

The illusory figures (Fig. 1) were the same as those used in Matsushita *et al*. (2013; 2014). The figure consisted of circular patches (1.26°) of 12 rows and 12 columns on a blue square (CIE x = 0.16, y = 0.07; 13.0 cd/m^2^; 18.31° × 18.31°). Each patch was filled with a gradation of white (CIE x = 0.33, y = 0.37; 210 cd/m^2^) to magenta (CIE x = 0.28, y = 0.28; 98.7 cd/m^2^). In the horizontal illusion stimulus (Fig. 1a), the direction of the white to magenta gradation was left to right or right to left. The 12 rows were split into six pairs. The gradation direction was identical within each pair and opposite that of the adjacent pairs. The vertical illusion stimulus (Fig. 1b) was identical to the horizontal illusion stimulus but rotated 90°. The stimulus was presented via a screen on a white background.

Eye motion was recorded with a Tobii X120 eye-tracking device (Tobii Technology, Stockholm, Sweden) directly below the screen at a sampling rate of 60 Hz. A charge-coupled device (CCD) camera was located just below the eye-tracking device; thus, the experimenter was able to observe, unseen by the infants, from a separate booth.

### Procedure

The infants sat on their parents’ laps and observed the liquid crystal display (LCD) screen (GW2255; 21.5 inch; 1920 × 1080 pixels; BenQ Corporation). The screen was at a distance of approximately 60 cm from the infants’ eyes. During the experiment, the parents were instructed to close their eyes and remain as still and quiet as possible.

In each trial, the experimenter confirmed that the infant was looking at the screen and presented the illusory stimulus for 10 s. Since there were two presentations for the horizontal and vertical stimuli, there were four trials in total. The trial order was randomized.

### Data availability

The datasets generated during and/or analyzed during the current study are available from the corresponding author on reasonable request.

## Results

We analyzed only the data that were recorded during the presentation of the illusory stimulus. We first assessed the quality of the eye-tracking data using Tobii’s ValidityLeft and ValidityRight outputs. The results revealed that the eye-tracking system correctly captured both eyes in 62.5% (*SD* = 16.4) of frames on average; we used only those frames for the analysis. Further analyses did not include the data from three infants for whom fewer than 40% of frames were valid.

**Figure 2:**
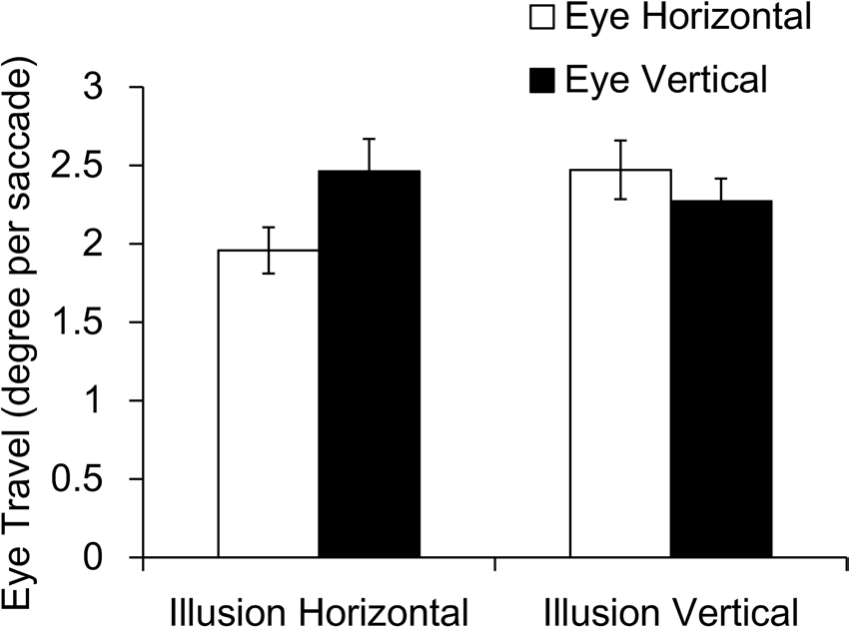
Eye movement results. Error bars represent the standard error of the mean.

Next, we computed the eye motion path by connecting each fixation to the next one. We used the median of the coordinates for each fixation, which was identified by Tobii Studio’s (version 3.0.9.425) default criterion. There were 14.2/trial (*SD* = 6.7) fixation-to-fixation paths (saccades) on the horizontal stimulus and 15.6/trial (*SD* = 9.4) on the vertical stimulus. Next, we resolved each path into horizontal and vertical vectors, which were the distances that the eyes traveled (Fig. 2). Finally, we analyzed horizontal and vertical travel distance per saccade.

Two-way ANOVA with stimulus direction and eye motion direction as independent factors revealed a significant interaction, *F*(1, 17) = 7.773, *p* = 0.013, η_p_^2^ = 0.314. To examine the detailed influence of the factors within each condition, we performed post hoc comparisons. The simple effect indicated that the vertical travel distance was longer than the horizontal travel distance for the horizontal stimulus, *F*(1, 34) = 4.433, *p* = 0.043, η_p_^2^ = 0.115. In addition, the horizontal travel distance was longer in the vertical stimulus condition than in the horizontal stimulus condition, *F*(1, 34) = 8.197, *p* = 0.007, η_p_^2^ = 0.194. To confirm that the bias of those travel distances reflected the saccade direction, we also computed the angle of each motion path from the ratio of vertical and horizontal distances; since both distances were unsigned values, the possible range was 0° to 90°. The mean angle in the horizontal stimulus condition (*M* = 53.58°, *SD* = 11.28) was significantly larger than 45°, *t*(17) = 3.228, *p* = 0.005, while the direction in the vertical stimulus condition (*M* = 47.04°, *SD* = 9.40) was not, *t*(17) = 0.919, *p* = 0.371. This vertical dominant eye motion in the horizontal condition was consistent with the results of ANOVA.

## Discussion

The results demonstrated that infants produced more orthogonal than parallel eye movement with respect to gradation direction. The eye movement pattern of the infants was similar to that of adults^3,4^. To our knowledge, this study is the first to reveal the detailed features of infants’ eye movements while observing gradation-motion illusion stimuli. The dominance of vertical eye motion in the horizontal illusion stimulus is a distinctive pattern of this illusion. This result occurred only for our illusion perception, because a previous infant study showed the dominance of horizontal eye motion in natural scene perception^5^. Based on these findings, we consider that infants perceived the figure in the same manner as adults.

The findings may be associated with the development of response timing of the infant visual nervous system to brightness and/or contrasts. At least in adults, the visual nervous system responds quicker to a high-contrast stimulus than to a low-contrast stimulus. Some recent studies assert that those spatio-temporal differences in the response to the gradation pattern illusion evokes a motion signal similar to apparent movement^6^. Thus, our finding suggests similarities between the nervous system of infants and adults in terms of the time course of its response.

Several limitations concerning whether the infants perceived illusory motion require consideration. It is possible to interpret the results as the infants merely moving their eyes to cross the gradation direction, regardless of the illusion. However, it is not reasonable to suppose that mere gradation without illusion overcomes the common bias of making horizontal eye motion^5^ and produces vertical eye motion. Thus, it is natural to think that the infant’s eye motion observed in this study is induced by the illusion.

It is unclear whether infants “intend” to move their eyes to enhance the magnitude of the illusory motion. Because it is difficult to examine a subject’s intention without using language, we cannot conclude that the infants intentionally moved their eyes to enjoy the illusion. However, the results revealed a clear interaction between the infants’ perception of the gradation-motion illusion and their eye movements, as shown by the non-random eye movement pattern.
